# Inhibition of the BET family reduces its new target gene *IDO1* expression and the production of l-kynurenine

**DOI:** 10.1038/s41419-019-1793-9

**Published:** 2019-07-19

**Authors:** Chang-Qing Tian, Lin Chen, Hua-Dong Chen, Xia-Juan Huan, Jian-Ping Hu, Jing-Kang Shen, Bing Xiong, Ying-Qing Wang, Ze-Hong Miao

**Affiliations:** 1 Division of Anti-Tumor Pharmacology, State Key Laboratory of Drug Research, Shanghai Institute of Materia Medica, Chinese Academy of Sciences, 201203 Shanghai, China; 2 University of Chinese Academy of Sciences, No.19A Yuquan Road, 100049 Beijing, China; 3 Department of Medicinal Chemistry, State Key Laboratory of Drug Research, Shanghai Institute of Materia Medica, Chinese Academy of Sciences, 201203 Shanghai, China; 4 Open Studio for Drugability Research of Marine Natural Products, Pilot National Laboratory for Marine Science and Technology (Qingdao), 266237 Shandong, China

**Keywords:** Cancer immunotherapy, Immunosuppression

## Abstract

The bromodomain and extra terminal domain (BET) family members, including BRD2, BRD3, and BRD4, act as epigenetic readers to regulate gene expression. Indoleamine 2,3-dioxygenase 1 (IDO1) is an enzyme that participates in tumor immune escape primarily by catalyzing tryptophan to l-kynurenine. Here, we report that *IDO1* is a new target gene of the BET family. RNA profiling showed that compound 9, a new BET inhibitor, reduced *IDO1* mRNA up to seven times in Ty-82 cells. IDO1 differentially expressed in tumor cells and its expression could be induced with interferon gamma (IFN-γ). BET inhibitors (ABBV-075, JQ1, and OTX015) inhibited both constitutive and IFN-γ-inducible expression of *IDO1*. Similarly, reduction of BRD2, BRD3, or BRD4 decreased *IDO1* expression. All these BET family members bound to the *IDO1* promoter via the acetylated histone H3. JQ1 led to their release and reduced enrichment of RNA polymerase II (Pol II) on the promoter. IFN-γ increased the binding of BRD2, BRD3, BRD4, and Pol II on the *IDO1* promoter by increasing the acetylation of histone H3, which could be prevented by JQ1 partially or even completely. Furthermore, both JQ1 and OTX015 decreased the production of l-kynurenine. The combination of BET inhibitors with the IDO1 inhibitor further reduced l-kynurenine, though only marginally. Importantly, the BET inhibitor ABBV-075 significantly inhibited the growth of human Ty-82 xenografts in nude mice and reduced both protein and mRNA levels of *IDO1* in the xenografts. This finding lays a basis for the potential combination of BET inhibitors and IDO1 inhibitors for the treatment of IDO1-expressing cancers.

## Introduction

The bromodomain and extra terminal domain (BET) family consists of BRD2, BRD3, BRD4, and BRDT. Except for BRDT that is restricted to the testes, the other BET family members are extensively expressed in human tissues and act as acetylation readers to activate polymerase II (Pol II) by recruiting the transcription elongation factor b Cyclin T1/CDK9 complex^1^. BET proteins are overexpressed in cancer and promote the expression of some key oncogenes, such as *MYC* and *BCL2*^1,2^. Thus, BET inhibitors, including ABBV-075, OTX015, and GSK525762, have entered into clinical trials, and more inhibitors have been reported as new anticancer drugs^1,3–6^. However, BET inhibitors as a monotherapy do not seem to produce enough ideal therapeutic activities, particularly in solid cancers. Recently, the BET family has been reported to regulate the expression of PD-L1^7,8^ and several DNA repair factors^9^, which can be inhibited by BET inhibitors. These findings offer a possibility that BET inhibitors are combined with anti-PD-L1 therapeutics or PARP inhibitors for cancer therapy, thus greatly expanding the potential therapeutic scope of BET inhibitors^7,9^.

In preliminary investigations, it was found that compound 9, a new BET inhibitor, which shows good in vitro and in vivo antitumor activity and improved pharmacokinetic characteristics^6^, could reduce the expression of indoleamine 2,3-dioxygenase (IDO, also known as IDO1). IDO1 is an initial rate-limiting enzyme of the l-kynurenine pathway. It catalyzes the metabolism of tryptophan into a variety of metabolites, including l-kynurenine and 3-hydroxyanthranilate^10^. These metabolites, especially l-kynurenine, promote naive CD4^+^ T cells to differentiate into CD4^+^CD25^+^Foxp3^+^-suppressive regulatory T cells (Tregs)^11^, induce cell cycle arrest and apoptosis of effector T (Teff) cells^12^, and inhibit the function of natural killer cells^13^. In addition, the other two tryptophan dioxygenases, i.e., IDO2 and tryptophan 2,3-dioxygenase (TDO, encoded by *TDO2*), exert very similar activities^14^. IDO1 has more extensive substrates and higher affinity for its substrates than both IDO2 and TDO^15^. IDO1 has been considered to be an immune checkpoint protein, just like programmed death-1 (PD-1), programmed death-ligand 1 (PD-L1), and cytotoxic T-lymphocyte-associated molecule-4^16^. IDO1 is constitutively expressed in more than half of human cancers, such as ovarian and colorectal cancer^17^. Moreover, the *IDO1* expression can be induced by interferon gamma (IFN-γ)^18^ by increasing the phosphorylation of the signal transducer and activator of transcription 1 (STAT1)^19^ and regulated by STAT3^20,21^. Therefore, IDO1 has been recognized as a promising anticancer target. IDO1 inhibitors are undergoing clinical trials for cancer therapy^10^.

In this study, we validated that BET inhibitors decreased the constitutive and IFN-γ-induced expression of *IDO1* at protein and mRNA levels. The downregulation of the BET family members, including BRD2, BRD3, and BRD4, rather than STAT1, elicited similar effects as the BET inhibitors. Mechanistic investigations showed that all BRD2, BRD3, and BRD4 bound to the acetylated histone H3 at the *IDO1* promoter, which was increased by IFN-γ but suppressed by BET inhibitors. The results indicate that *IDO1* is a direct target gene of the BET family. Consequently, BET inhibitors led to decreased l-kynurenine production. Animal experiments showed that the BET inhibitor ABBV-075 inhibited the growth of human Ty-82 xenografts and reduced the *IDO1* expression in the xenografts. These findings offer a new target gene of the BET family proteins and are likely to expand the therapeutic applications of BET inhibitors to cancers that overexpress both the BET and IDO1 proteins.

## Results

### BET inhibitors reduce *IDO1* expression

**Fig. 1:**
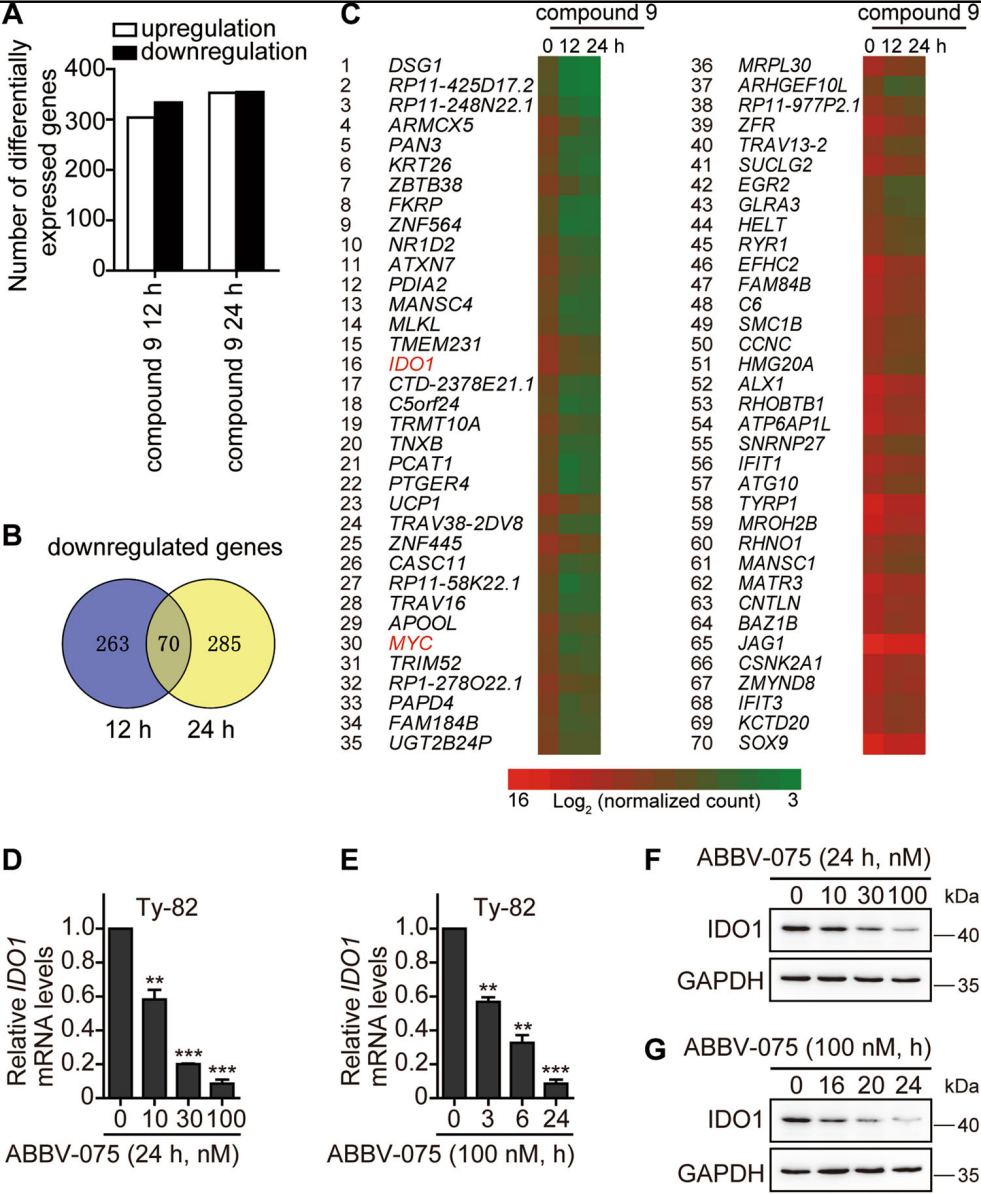
BET inhibitors reduce *IDO1* transcription. **a** The number of the significantly differentially expressed genes (fold change > 2, *p* < 0.05) in Ty-82 cells treated with 1 μM compound 9 for 12 or 24 h. **b** Genes of the differentially downregulated expression in the compound 9-treated Ty-82 cells were plotted as the Venn diagram to display the commonly downregulated genes. **c** Heatmaps of 70 common genes of the differentially downregulated expression in Ty-82 cells treated with compound 9 for 0, 12, or 24 h, ranked (1–70) by fold change from big to small after 24-h treatments. Also see Supplementary Table S1–3. **d**–**g** Ty-82 cells were treated with the BET inhibitor ABBV-075 at the indicated concentrations for the indicated time. The mRNA (**d**, **e**) and protein (**f**, **g**) levels of *IDO1* were determined by RT-qPCR and western blotting, respectively. Data were from three independent experiments, and if applicable, were expressed as mean ± SD (error bar); *p *<* 0.05; **p < 0.01; ***p < 0.001

Compound 9 is a new BET inhibitor with high selectivity toward the BET family and potent BRD4 inhibitory activity^6^. Its RNA profiling assayed by RNA sequencing (RNA-seq) showed that expression of 333 and 355 genes was downregulated in Ty-82 cells treated with this compound for 12 and 24 h, respectively (Fig. 1a, and Supplementary Tables S1 and S2). *MYC*, a well-known direct target gene of BRD4, appeared on the list of 70 common genes of downregulated expression at the two time points (Fig. 1b, c and Supplementary Tables S1–3). The 24-h treatment with compound 9 led to the decrease of *MYC* mRNA by more than five times. Moreover, the same treatment reduced the mRNA level of *IDO1* to a larger degree, up to seven times (Fig. 1c and Supplementary Table S2). IDO1 is an important anticancer target and its inhibitors are undergoing clinical trials. As a new BET inhibitor, many aspects of compound 9 are still unknown at present. Therefore, other well-known BET inhibitors, such as ABBV-075, JQ1, and OTX015, were used to continue the study to demonstrate the relationships between the BET family and *IDO1*. ABBV-075 is a potent BET inhibitor currently used in phase I clinical trials (ClinicalTrials.gov identifier NTC02391480). To verify the results about the *IDO1* expression from compound 9, we used ABBV-075 to treat Ty-82 cells. The data showed that ABBV-075 reduced the mRNA and protein levels of *IDO1* in concentration- and time-dependent manners (Fig. 1d–g).

### Different BET inhibitors inhibit the constitutive and inducible expression of *IDO1* in different tumor cells

**Fig. 2:**
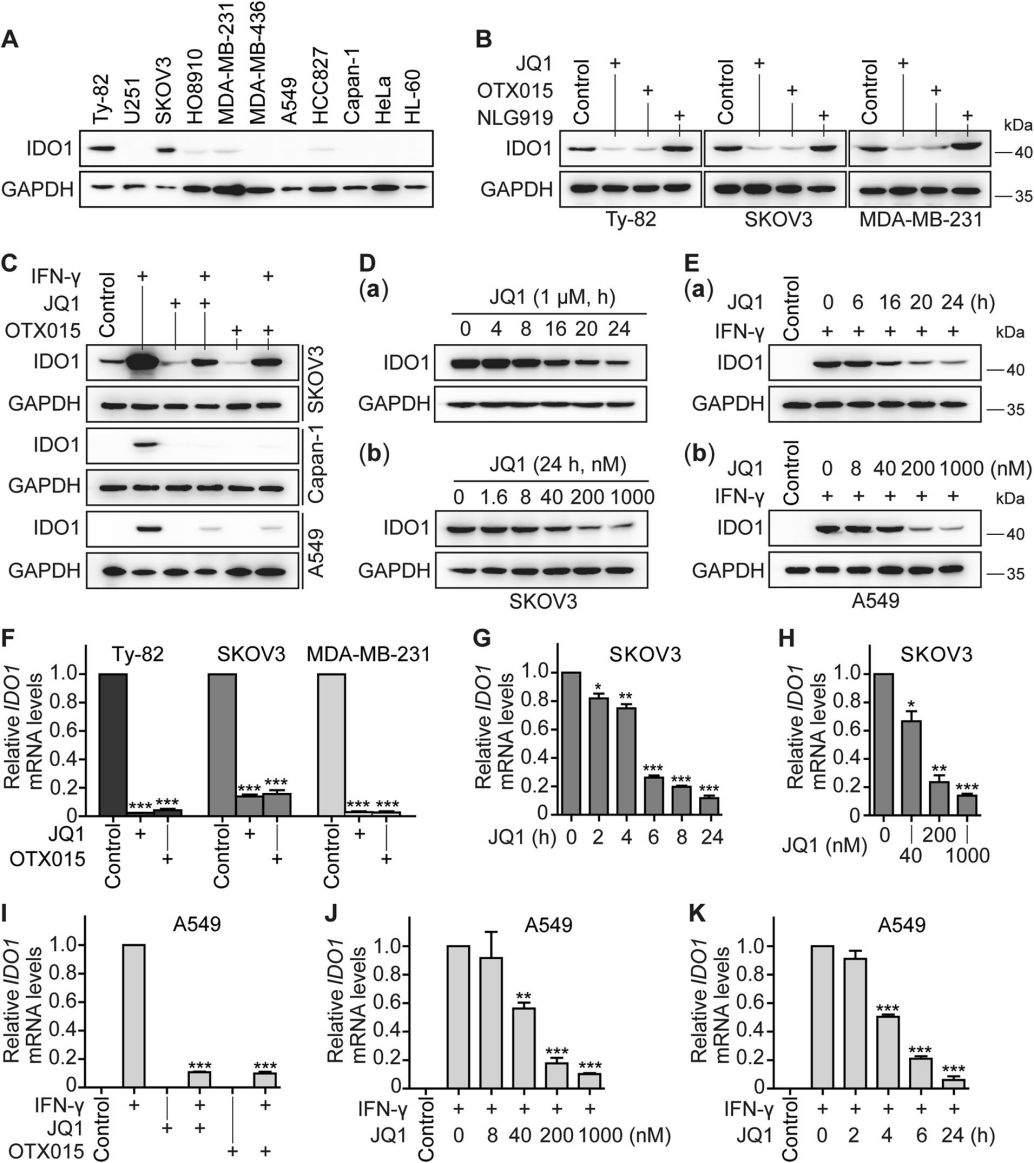
BET inhibitors reduce the constitutive and IFN-γ-induced expression of *IDO1*. The protein levels of IDO1 were detected by western blotting in human tumor cell lines derived from different tissues (**a**), in Ty-82, SKOV3, and MDA-MB-231 cells treated with JQ1, OTX015, or NLG919 at 1 μM for 24 h (**b**), in SKOV3, Capan-1, and A549 cells that were treated with IFN-γ (10 ng/ml), JQ1 (1 μM), OTX015 (1 μM), or their respective combinations for 24 h (**c**), in SKOV3 (**d**) and A549 (**e**) cells treated with JQ1 (1 μM) [**d**(**a**)] or IFN-γ (10 ng/ml, 24 h) plus JQ1 (1 μM) [**e**(**a**)] for the indicated time or with gradient concentrations of JQ1 [**d**(**b**)] or JQ1 plus IFN-γ (10 ng/ml) [**e**(**b**)] for 24 h. GAPDH was used as the loading control. The relative mRNA levels of *IDO1* were determined by RT-qPCR in Ty-82, SKOV3, and MDA-MB-231 cells treated with JQ1 or OTX015 at 1 μM for 24 h (**f**), in SKOV3 cells treated with JQ1 (1 μM) for the indicated time (**g**) or with gradient concentrations of JQ1 for 24 h (**h**), or in A549 cells treated with IFN-γ (10 ng/ml) plus JQ1 or OTX015 (1 μM) for 24 h (**i**), with IFN-γ (10 ng/ml) plus gradient concentrations of JQ1 for 24 h (**j**), or with IFN-γ (10 ng/ml, 24 h) plus JQ1 (1 μM) for the indicated time (**k**). All the data were from three independent experiments, and if applicable, were expressed as mean ± SD (error bar); *p < 0.05; **p < 0.01; ***p < 0.001; + , treated with the indicated drug

To know whether the *IDO1* reduction caused by ABBV-075 has the universality, we further detected the effect of different BET inhibitors on the expression of *IDO1* in different tumor cells. It was found that IDO1 was constitutively expressed in both Ty-82 and SKOV3 cell lines at high levels and in all HO8910, MDA-MB-231, and HCC827 cell lines at relatively low levels, but in the other six cell lines at almost non-detectable levels (Fig. 2a). The difference of the IDO1 protein levels in two breast cancer cell lines, MDA-MB-231 and MDA-MB-436 (Fig. 2a), suggests that the constitutive expression of cellular IDO1 might not be associated with the tissue origin of cancer cells. JQ1 and OTX015 are two widely used BET inhibitors. Separate treatments with them led to an apparent reduction of the IDO1 protein in all three cell lines (Ty-82, SKOV3, and MDA-MB-231) (Fig. 2b). However, under the same conditions, the IDO1 inhibitor NLG919 did not decrease the levels of the constitutive IDO1 (Fig. 2b). To investigate whether these BET inhibitors inhibit the inducible expression of *IDO1*, three cell lines that constitutively expressed a high level (SKOV3) or undetectable levels (Capan-1 and A549) of the IDO1 protein were used (Fig. 2a). Treatments with IFN-γ (10 ng/ml) resulted in dramatic enhancement of the IDO1 protein levels in all three cell lines. Both JQ1 and OTX015 almost completely removed this enhancement (Fig. 2c). Moreover, the BET inhibitor JQ1 reduced the levels of both the constitutive [Fig. 2d(a), (b)] and IFN-γ-induced [Fig. 2e(a), (b)] IDO1 protein in SKOV3 and A549 cell lines in concentration- and time-dependent manners.

These results suggest that BET inhibitors indeed inhibit the expression of *IDO1*. Therefore, it was further examined where different BET inhibitors cause the corresponding changes in the mRNA levels of *IDO1* in different tumor cells. Consistently, treatments with both JQ1 and OTX015 resulted in significant decreases at the mRNA levels of *IDO1* in Ty-82, SKOV3, and MDA-MB-231 cell lines (Fig. 2f). Such decreases showed typical concentration- and time dependency in JQ1-treated SKOV3 cells (Fig. 2g, h). In A549 cells that have no detectable *IDO1* mRNA, treatments with IFN-γ (10 ng/ml) induced the expression of the *IDO1* mRNA, which was suppressed significantly by both JQ1 and OTX015 (Fig. 2i). Similarly, JQ1 also inhibited the IFN-γ-induced *IDO1* mRNA in concentration- and time-dependent manners (Fig. 2j, k).

Therefore, our data indicate the universality of the reduction of the *IDO1* expression, both constitutive and IFN-γ-inducible, caused by BET inhibitors. Because the BET family members are the targets of BET inhibitors, these results suggest that those members are involved in the regulation of the *IDO1* expression.

### Reduction of BRD2, BRD3, or BRD4 rather than STAT1 leads to the decrease of the *IDO1* expression

**Fig. 3:**
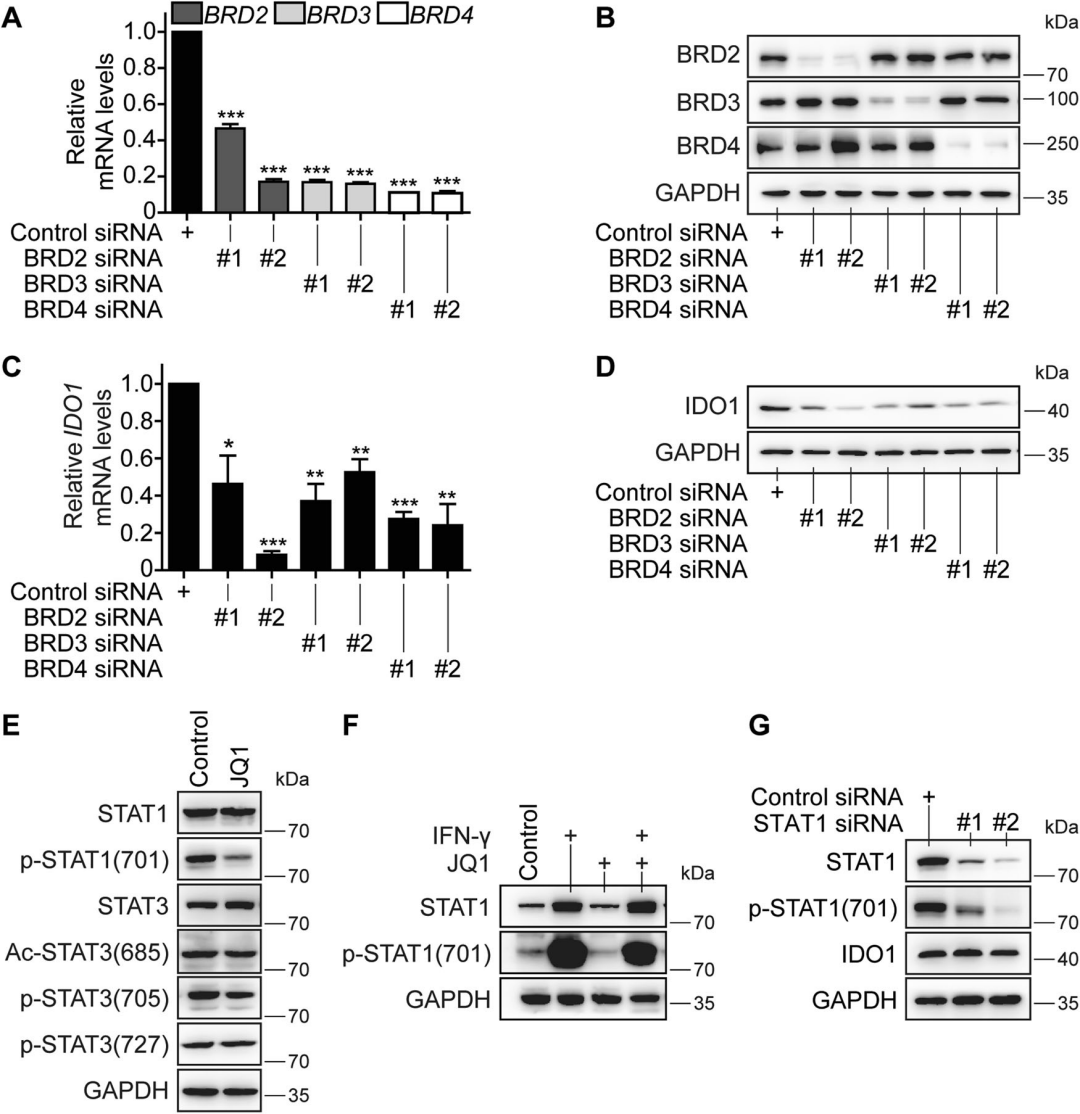
Reduced expression of the BET family members (BRD2, BRD3, and BRD4) but not STAT1 decreases the expression of *IDO1.* **a–d** SKOV3 cells were transfected with control siRNA or siRNA of two different sequences specific to *BRD2*, *BRD3*, or *BRD4*. After 48 h, mRNA (**a**, **c**) and protein (**b**, **d**) levels of *BRD2*, *BRD3*, *BRD4*, and *IDO1* were determined by RT-qPCR and western blotting, respectively. **e** SKOV3 cells were treated with or without 1 μM JQ1 for 24 h, and the protein levels of STAT1, p-STAT1(701), STAT3, Ac-STAT3(685), p-STAT3(705), and p-STAT3(727) were detected by western blotting. **f** SKOV3 cells were treated with IFN-γ (10 ng/ml), JQ1 (1 μM), or their combination for 24 h, and the protein levels of STAT1 and p-STAT1(701) were detected by western blotting. **g** SKOV3 cells were transfected with siRNA of two different sequences specific to *STAT1* or control siRNA for 48 h, and the protein levels of STAT1, p-STAT1(701), and IDO1 were detected by western blotting. Data were from three independent experiments, and if applicable, were expressed as mean ± SD (error bar); *p < 0.05; **p < 0.01; ***p < 0.001

The BET family has three known members, i.e., BRD2, BRD3, and BRD4^22^. To clarify their roles in regulating the *IDO1* expression, these members were separately knocked down with their respective small-interfering RNA (siRNA) in SKOV3 cells that constitutively express IDO1 (Fig. 2a). The results indicated that the corresponding RNA interference effectively lowered the mRNA (Fig. 3a) and protein (Fig. 3b) levels of *BRD2*, *BRD3*, and *BRD4*. Knockdown of any BET family member (*BRD2*, *BRD3*, or *BRD4*) did not affect the protein levels of other members (Fig. 3b). The reduced expression of each BET member resulted in the decrease of both the mRNA (Fig. 3c) and protein (Fig. 3d) levels of *IDO1* at comparable degrees. The results indicate that these BET members play almost equivalent and mutually independent roles in regulating the *IDO1* expression.

The *IDO1* expression is also regulated by STAT3 and STAT1 as previously reported^19–21^. The BET inhibitor JQ1 was indeed found to reduce the phosphorylation of STAT1 at Tyrosine 701 [p-STAT1(701)], though the drug did not obviously change the levels of total STAT1 and STAT3 proteins, and the acetylation or phosphorylation levels of STAT3 in SKOV3 cells (Fig. 3e). Moreover, JQ1 could prevent the IFN-γ-induced increase in p-STAT1(701), though only partly (Fig. 3f). However, interference with *STAT1* to reduce total STAT1 protein and p-STAT1(701) did not cause any apparent changes in the constitutively expressed IDO1 level in SKOV3 cells (Fig. 3g). These results indicate that the transcription factors STAT3 and STAT1 do not mediate the BET inhibitor-driven decrease of the constitutive *IDO1* expression.

### *IDO1* is a direct target gene of the BET family members

**Fig. 4:**
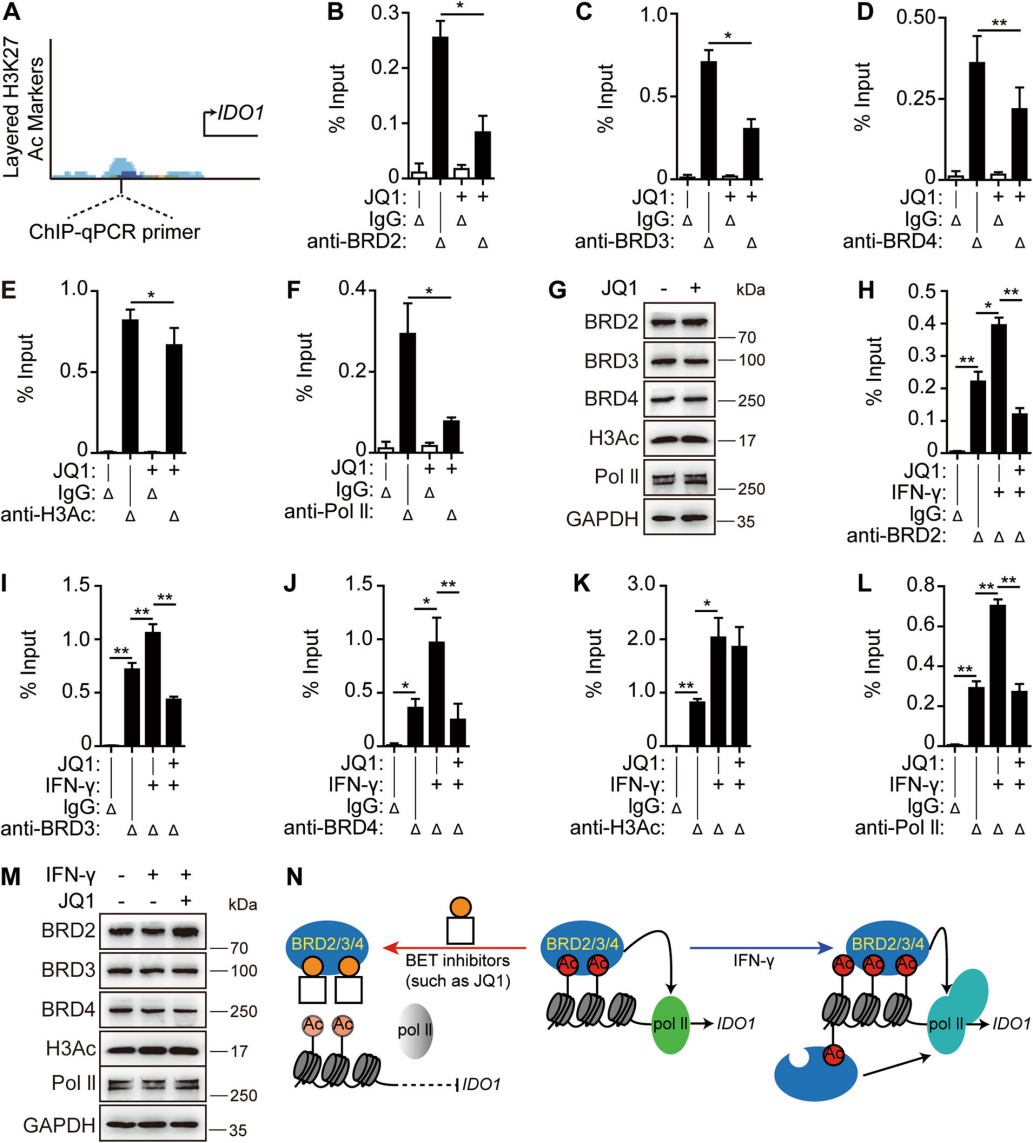
*IDO1* is a direct target gene of the BET family. **a** Schematic diagram of the ChIP-qPCR primer design. The peak shows the enrichment of the H3K27Ac on the *IDO1* promoter determined by the ChIP-seq assay in seven cell lines from the ENCODE website. **b–g** SKOV3 cells were treated with or without 1 μM JQ1 for 6 h. The cells were subjected to ChIP analyses with antibodies against BRD2 (**b**), BRD3 (**c**), BRD4 (**d**), H3Ac (**e**), or Pol II (**f**). The association of these proteins with the *IDO1* promoter was quantified by qPCR. An isotype-matched IgG was used as the negative control. The protein levels were determined by western blotting (**g**). **h–m** SKOV3 cells were treated with IFN-γ (10 ng/ml), JQ1 (1 μM), or their combination for 6 h. The cells were subjected to ChIP analyses with the indicated antibodies, and the association of BRD2 (**h**), BRD3 (**i**), BRD4 (**j**), H3Ac (**k**), or Pol II (**l**) with the *IDO1* promoter was quantified by qPCR. The protein levels were determined by western blotting (**m**). **n** A model for the *IDO1* transcription regulated by BET family members. Data were from three independent experiments, and if applicable, were expressed as mean ± SD (error bar); *p < 0.05; **p < 0.01; + , treated with JQ1 or IFN-γ; Δ, detected with the corresponding antibody. Also see Supplementary Fig. S1

BRD4 binds promoters and enhancers^23^, and one of the prominent markers of those enhancers is the acetylation of H3K27^24^. According to the ENCODE database (https://www.encodeproject.org/), the *IDO1* promoter region has a remarkable H3K27 acetylation enrichment (Fig. 4a). It was thus examined whether *IDO1* is a direct target gene of the BET family. Chromatin immunoprecipitation analyses showed that treatments with JQ1 led to the significant release of BRD2 (Fig. 4b), BRD3 (Fig. 4c), and BRD4 (Fig. 4d) from the *IDO1* promoter. Unexpectedly, JQ1 slightly reduced the level of acetylated histone H3 (H3Ac) at the same region (Fig. 4e). This might be an effect secondary to the decreased binding of the BET family members to the *IDO1* promoter. Consistent with the JQ1-mediated inhibition of constitutive *IDO1* expression (Fig. 2b, f), JQ1 led to reduced enrichment of Pol II on the *IDO1* promoter (Fig. 4f). However, the treatment with JQ1 did not reduce the cellular total protein levels of BRD2, BRD3, BRD4, H3Ac, or Pol II (Fig. 4g).

When examining the IFN-γ-inducible *IDO1* expression, it was found that the treatment with IFN-γ led to a significant increase in the BRD2, BRD3, and BRD4 enrichment on the *IDO1* promoter (Fig. 4h–j). The co-treatment with IFN-γ and JQ1 completely ablated this increase, even decreasing the BRD2, BRD3, and BRD4 enrichment to a level lower than that at the basal state (Fig. 4h–j). As expected, the IFN-γ treatment significantly enhanced the acetylation levels of histone H3 (Fig. 4k) and the Pol II enrichment (Fig. 4l) on the *IDO1* promoter. JQ1 removed the IFN-γ-stimulating enhancement of the Pol II enrichment totally but the H3 acetylation only partly (Fig. 4k, l). Notably, the treatments with either IFN-γ alone or IFN-γ plus JQ1 did not cause an obvious change in the protein levels of BRD2, BRD3, BRD4, H3Ac, or Pol II (Fig. 4m).

The human α-satellite repeat was used as a putative negative control in chromatin immunoprecipitation analyses^25^ to examine the specificity of the binding of the detected proteins to the *IDO1* promoter region (Supplementary Fig. S1). The data showed that all detected proteins (BRD2, BRD3, BRD4, H3Ac, and Pol II) were enriched on the *IDO1* promoter region at significantly higher levels than on the α-satellite repeat in the intact cells. Moreover, drug treatments did not cause obvious changes in the binding levels of these proteins on the α-satellite repeat, a sharp contrast to the situation pertaining to the *IDO1* promoter (Fig. 4b–f, h–l and Supplementary Fig. S1A–J).

These results reveal that the BET family members specifically bind to the acetylated histone H3 on the *IDO1* promoter region, which might facilitate both constitutive and IFN-γ-inducible transcription of the *IDO1* gene. This process can be suppressed by BET inhibitors (Fig. 4n), suggesting that *IDO1* is a direct target gene of the BET family.

### BET inhibitors reduce the production of l-kynurenine

**Fig. 5:**
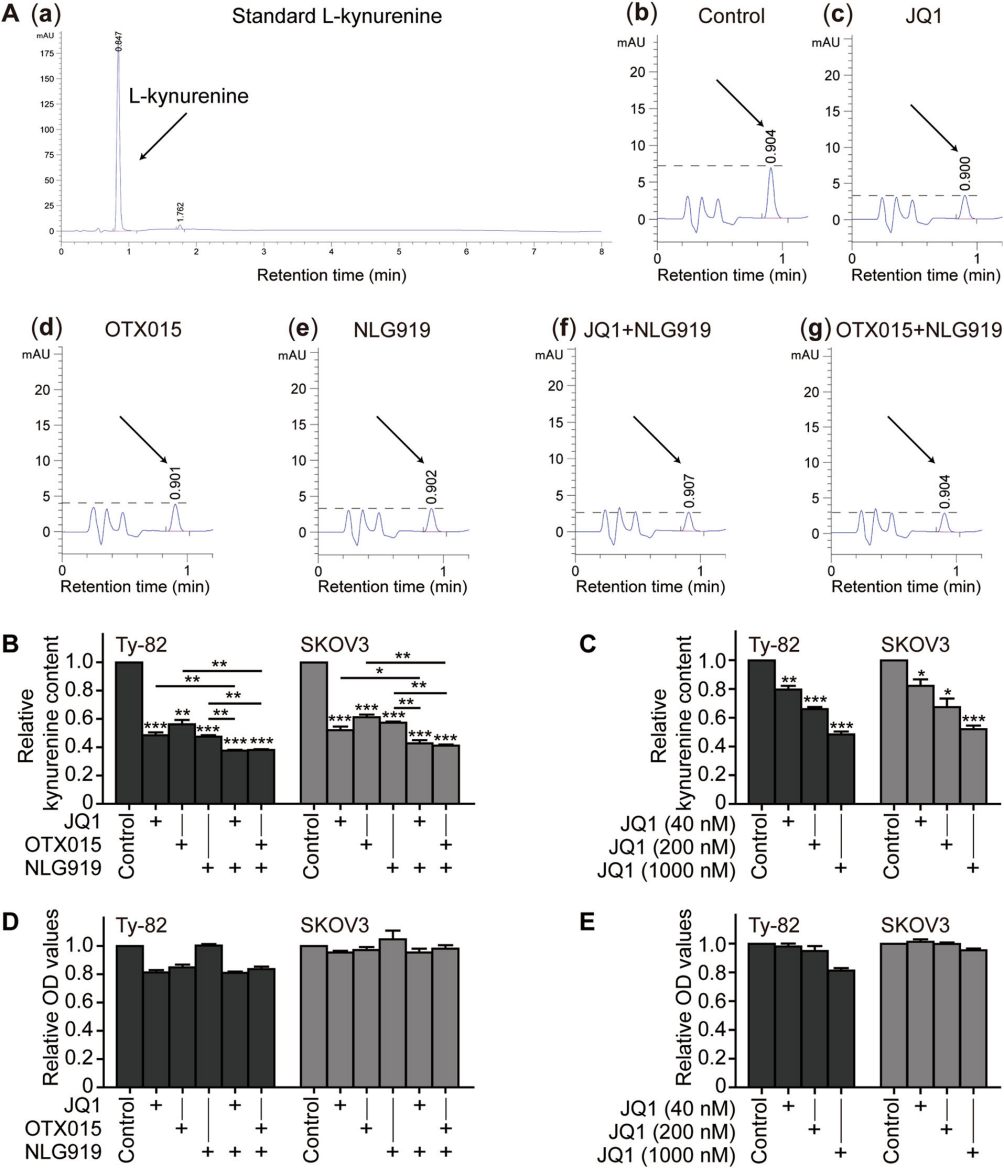
BET inhibitors decrease the production of l-kynurenine in tumor cells. **a** HPLC chromatograms of l-kynurenine for the reference (**a**) and in the medium of the cultured Ty-82 cells exposed to 0.1% DMSO (**b**), 1 μM JQ1 (**c**), 1 μM OTX015 (**d**), 1 μM NLG919 (**e**), 1 μM JQ1 plus 1 μM NLG919 (**f**), or 1 μM OTX015 plus 1 μM NLG919 (**g**) for 24 h. The arrowhead indicates the peak of l-kynurenine. **b**, **c** The relative l-kynurenine content (normalized ratio of area under HPLC curve) was determined by HPLC in the medium of the cultured Ty-82 and SKOV3 cells treated with the indicated compounds at 1 μM (**b**) or gradient concentrations of JQ1 (**c**) for 24 h. **d**, **e** Cell proliferation (expressed as normalized relative OD value) was measured by the SRB assay after 24-h exposure of the cells to the indicated compounds at 1 μM (**d**) or gradient concentrations (**e**) of JQ1. Data were from three independent experiments, and if applicable, were expressed as mean ± SD (error bar); *p < 0.05; **p < 0.01; ***p < 0.001; +, treated with the indicated drug

IDO1 can catalyze tryptophan into l-kynurenine^10^. As expected, the IDO1 inhibitor NLG919 significantly reduced the production of l-kynurenine in human cancer Ty-82 and SKOV3 cells [Fig. 5a(a–e), b]. Notably, both the BET inhibitors, JQ1 and OTX015, also decreased the production of l-kynurenine, and their reduction degrees were equivalent to that caused by the IDO1 inhibitor NLG919 [Fig. 5a(a–e), b]. Moreover, the effect caused by the BET inhibitor JQ1 showed an apparent concentration dependency (Fig. 5c). Compared with the BET inhibitors or the IDO1 inhibitor alone, the combination of BET inhibitors with the IDO1 inhibitor further reduced the production of l-kynurenine [Fig. 5a(a–g), b], though only marginally. In contrast, neither the IDO1 inhibitor nor the BET inhibitors greatly affected the proliferation of the cancer cells exposed to the same treatments (Fig. 5d, e), indicating that the reduction of the l-kynurenine production caused by these inhibitors is independent of their proliferative inhibition. The results indicate that by inhibiting the *IDO1* expression, the BET inhibitors elicit similar effects on l-kynurenine as the IDO1 inhibitor does.

**Fig. 6:**
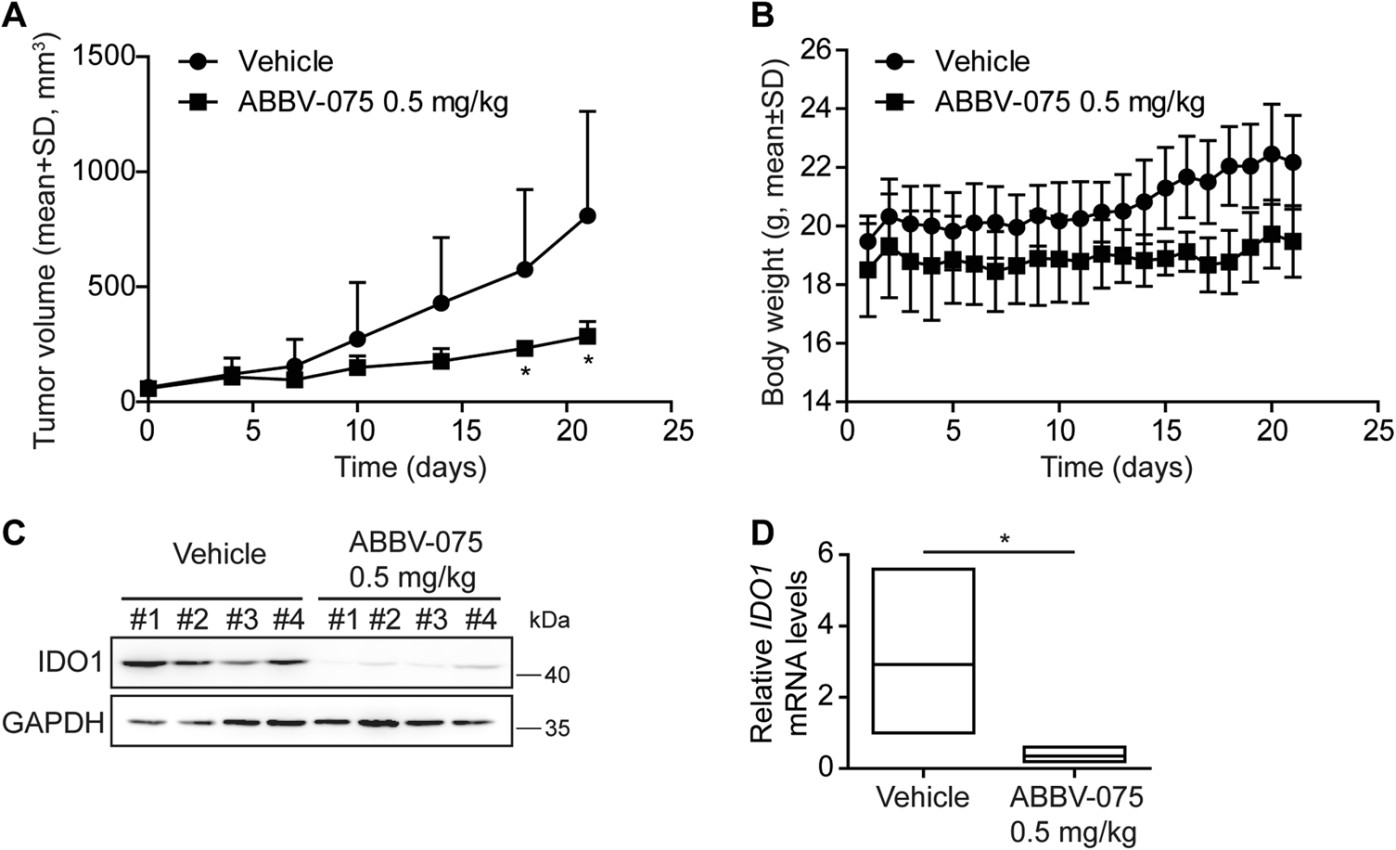
ABBV-075 decreases *IDO1* expression in vivo. **a** Changes in the mean of tumor volume of human Ty-82 xenografts in Balb/c nude mice following the treatment with the BET inhibitor ABBV-075. *p < 0.05 (ABBV-075 group vs. vehicle group). Formulation for ABBV-075: 1% dimethylacetamide and 5% Solutol SH15. **b** Changes in body weight of Balb/c nude mice harboring Ty-82 xenografts. **c** The IDO1 protein levels of human Ty-82 xenografts after the 21-day ABBV-075 treatment. #1, #2, #3, and #4 stand for different mice. **d** The relative *IDO1* mRNA levels in human Ty-82 xenografts after the 21-day ABBV-075 treatment (upper line: highest value; middle line: mean; lower line: lowest value)

Nevertheless, it is worth noting that BET inhibition (by using BET inhibitors or siRNA interference) did not completely eliminate the expression of *IDO1* at both protein and mRNA levels or the production of l-kynurenine (Figs. 1–6).

### The BET inhibitor ABBV-075 decreases the *IDO1* expression in vivo

To examine whether BET inhibitors can reduce the expression of *IDO1* in vivo, Balb/c nude mice bearing human IDO1-expressing Ty-82 xenografts were treated with ABBV-075 at 0.5 mg/kg daily. The result showed that ABBV-075 significantly inhibited the tumor growth (Fig. 6a) and was well tolerated (Fig. 6b). Simultaneously, ABBV-075 reduced the expression of *IDO1* at both protein and mRNA levels in the xenografts (Fig. 6c, d). The data indicate that BET inhibitors indeed reduce the expression of *IDO1* both in vivo and in vitro, further suggesting that such a reduction might be used as a pharmacodynamic marker to monitor the pharmacological effects of BET inhibitors on IDO1-expressing tumors.

## Discussion

The clinical development of BET inhibitors as an anticancer monotherapy has not progressed smoothly due to relatively low responses and high relapses during their treatment^1^. Recently, IDO1 inhibitors have also suffered their failure in the first phase III clinical trial^26^. Therefore, determining the proper approaches to change the unfavorable situations to both inhibitors becomes a critical concern for their future development. In this study, it was found that BET inhibitors reduced, but did not eliminate, the constitutive and IFN-γ-inducible expression of *IDO1* and the production of l-kynurenine. It was further demonstrated that *IDO1* was a new direct target gene of the BET family members including BRD2, BRD3, and BRD4. These findings offer new insights into the development of both BET and IDO1 inhibitors.

The BET family proteins are the epigenetic readers that regulate the expression of genes including those encoding transcription factors and other proteins^1,27^. A general mode of the BET proteins including BRD2, BRD3, and BRD4 to regulate gene transcription is by co-occupying the acetylated histones at the promoters of their target genes and Pol II, and by removing such occupation, BET inhibitors inhibit gene transcription^27^. Data have consistently shown that BRD2, BRD3, and BRD4 directly bind to the acetylated H3K27 sites at the promoter of *IDO1* and Pol II. Their binding is significantly reduced in response to treatment with JQ1. Moreover, knockdown of each of those BET family members led to a similar reduction in the *IDO1* expression. However, the pan-BET inhibitors ABBV-075, JQ1, or OTX015 did not eliminate either constitutive or IFN-γ-induced *IDO1*, even though they can inhibit all BRD2, BRD3, and BRD4^28^. These results indicate that BRD2, BRD3, and BRD4 play an overlapping role in regulating the *IDO1* transcription in IDO1-expressing tumors.

Different from the majority of the previously reported proteins encoded by the BET-regulated genes^1,7,27,29^, IDO1 mediates metabolic immune escape in cancer^26^. This enzyme by its catalytic activity converts tryptophan into l-kynurenine. The latter binds to the aryl hydrocarbon receptor (AHR) to elicit immunosuppressive effects. Moreover, the binding of IDO1 by its nonenzymatic activity to AHR is necessary for the immunosuppressive effects. In addition, the l-kynurenine-activated, AHR-mediated gene expression further induces the expression of *IDO1*, forming a positive feedback loop^10,30^. IDO1 inhibitors, in particular the selective ones, only inhibit its catalytic activity but do not impair its nonenzymatic activity, and l-kynurenine can also be produced by the catalysis of IDO2 or TDO. These might be some possible reasons that the IDO1 inhibitor epacadostat recently suffered failure in its phase III clinical trial^31^. In contrast, BET inhibitors such as JQ1, OTX015, and ABBV-075 reduce both the constitutive and inducible expression of *IDO1*. The combination of BET inhibitors with IDO1 inhibitors further enhances the inhibitory effects on l-kynurenine production though only marginally. One possible reason for this marginal effect might be that the reduction of IDO1 caused by the BET inhibitors decreases the effect of the IDO1 inhibitor via inhibiting IDO1. Therefore, the combination of BET inhibitors is likely to improve the immune checkpoint inhibitory activity of IDO1 inhibitors by further reducing the l-kynurenine production, decreasing the binding of IDO1 to AHR and blocking the IDO1–AHR feedback. Actually, our data show that BET inhibitors inhibit tumor growth and simultaneously reduce the *IDO1* expression in the xenografts. However, although IDO1 and its metabolites have been reported to directly promote the cell proliferation and tumorigenesis and inhibit apoptosis in colon cancer cells^32–34^, our current data could still not establish a direct causal link between the tumor regression and the reduced IDO1 expression following the treatment with BET inhibitors, which requires additional investigations. Nevertheless, those consistent in vitro and in vivo data suggest that the reduction of the *IDO1* expression responding to the treatment of BET inhibitors might be used as a pharmacodynamic marker to monitor the pharmacological effects of BET inhibitors on IDO1-expressing tumors. Furthermore, BET inhibitors have been demonstrated to exert their anticancer effects via other approaches, such as inhibiting the expression of oncogenes including *MYC*^1^.

In contrast to the extensive expression of the BET family members BRD2, BRD3, and BRD4^1^, the expression of IDO1 varies in different tissues^10,30^. Our data also show that among the 11 tested tumor cell lines, only five constitutively express IDO1. When these two types of inhibitors are combined for either preclinical or clinical study, the high co-expression of both BET and IDO1 proteins in cancer cells is required. These findings indicate that more investigations are warranted, particularly considering the present situations of both BET and IDO1 inhibitors.

## Materials and methods

### Cell culture

Ty-82 cells were purchased from the Japanese Collection of Research Bioresources Cell Bank (Osaka, Japan). HL-60, U251, HO8910, and HCC827 cells were obtained from the Cell Bank of the Chinese Academy of Sciences Type Culture Collection (Shanghai, China). SKOV3, MDA-MB-231, MDA-MB-436, A549, Capan-1, and HeLa cells were obtained from the ATCC (Manassas, VA, USA). All cells were cultured in the corresponding conditions recommended by the suppliers.

### Chemicals, reagents, and antibodies

BET inhibitors JQ1 (S7110) and OTX015 (S7360) and the IDO1 inhibitor NLG919 (S7111) were purchased from Selleck Chemicals (Houston, TX, USA). The BET inhibitor ABBV-075 (HY-100015) was obtained from Medchem Express (Monmouth Junction, NJ, USA). N,N-Dimethylacetamide was purchased from Sigma (CA, USA). Solutol SH15 was obtained from BASF Chemical Company (Ludwigshafen, Germany). The BET inhibitor compound 9 was prepared according to the reported procedure with the purity of over 99% determined by the high performance liquid chromatography (HPLC)^6^. l-kynurenine (MB5637) was obtained from Dalian Mei Lun Biotechnology Co., Ltd. (Dalian, China). The anti-IDO (#86630), anti-BRD2 (#5848), anti-BRD4 (#13440), anti-Rpb1 (the largest subunit of Pol II) NTD (#14958), anti-STAT1 (#14994), anti-phospho-STAT1(Tyr701) (#9167), anti-STAT3 (#9139), anti-phospho-STAT3 (Tyr705) (#9145), anti-phospho-STAT3 (Tyr727) (#9134), and anti-acetyl-STAT3 (Lys685) (#2523 L) antibodies were purchased from Cell Signaling Technology (Boston, MA, USA). Anti-GAPDH (AF0006) antibody was purchased from Beyotime Biotechnology (Shanghai, China), anti-acetyl histone H3 (06-599) antibody was from Millipore (MA, USA), and anti-BRD3 (61489) antibody was from Active Motif (Carlsbad, CA, USA). Recombinant human IFN-γ (300–02) was obtained from PeproTech (Rocky Hill, NJ, USA). The secondary antibodies HRP-conjugated goat anti-rabbit (111–035–003) and goat anti-mouse (115-035-003) were purchased from Jackson ImmunoResearch Laboratories, Inc. (West Grove, PA, USA).

### RNA sequencing (RNA-seq)

Ty-82 cells were plated into 10-cm cell culture dishes and incubated for 24 h, and then 1 μM compound 9 was added and treated for 0, 12, or 24 h. Cells were harvested in TRIzol (Life Technologies; CA, USA) and extracted for RNA. Each mRNA was enriched with oligo(dT) and fragmented into small fragments. These fragments underwent reverse transcription, 3′ and 5′ adaptors ligation, PCR amplification, library construction, RNA sequencing using HiSeq2500 (Illumina Inc.; San Diego, CA, USA), and data analysis at Genergy Biotechnology Co., Ltd. (Shanghai, China).

### Small RNA interference

All small siRNA, including control siRNA, were purchased from Genepharma (Shanghai, China). Transfection was conducted using Lipofectamine RNAiMAX (13778075) (Invitrogen; Carlsbad, CA, USA) following the manufacturer’s guidance. The sequences of siRNA duplexes were as follows: *BRD2*: 5′-CACGAAAGCUACAGGAUGU-3′ (#1) and 5′-GGGCCGAGUUGUGCAUAUA-3′ (#2); *BRD3*: 5′-AGTGAGTGTATGCAGGACTTCAACACCAT-3′ (#1) and 5′-CGGCUGAUGUUCUCGAAUU-3′ (#2); *BRD4*: 5′-CUCCCUGAUUACUAUAAGATT-3′ (#1) and 5′-GCACAAUCAAGUCUAAACUTT-3′ (#2); *STAT1*: 5′-GCUGGAUGAUCAAUAUAGUTT-3′ (#1) and 5′-GCGUAAUCUUCAGGAUAAUTT-3′ (#2); and the control siRNA: UUCUCCGAACGUGUCACGUdTdT.

### Real-time quantitative PCR (RT-qPCR)

Total RNA was extracted with TRIzol and turned into cDNA by reverse transcription with PrimeScript™ RT Master Mix (Perfect Real Time) (RR036A) (TaKaRa; Kusatsu, Shiga, Japan). The reaction of 10 μl required 250 ng total RNA. The resulting cDNA was detected by RT-qPCR using SYBR® Premix ExTaq™ (Tli RNaseH Plus) (RR820A) (TaKaRa; Kusatsu, Shiga, Japan). The primers for human *IDO1*, *BRD2, BRD3, BRD4*, and *β-actin* were as follows: *IDO1*: 5′-TTCAGTGCTTTGACGTCCTG-3′ (forward) and 5′-TGGAGGAACTGAGCAGCAT-3′ (reverse); *BRD2*: 5′-GGCTTGGCCAAATCGTCTTC-3′ (forward) and 5′-TCATGTACTGCCCGAAGCTG-3′ (reverse); *BRD3*: 5′-CTGAAACCCACCACTTTGCG-3′ (forward) and 5′-CTGTTTCTTCCCGCTTGCTG-3′ (reverse); *BRD4*: 5′-CACCCAGCACCAGAGAAGAG-3′ (forward) and 5′-GTCCATGGGGTGCTTGATGA-3′ (reverse); and *β-actin*: 5′-ATCGTGCGTGACATTAAGGAGAAG-3′ (forward) and 5′-AGGAAGGAAGGCTGGAAGAGTG-3′ (reverse).

### Western blotting

Cells were plated into 6-well plates and incubated for 24 h. Then, the cells were treated with the indicated drugs, collected, and boiled for 10 min at 100 °C. Western blotting was done as previously reported^6^. GAPDH was used as the loading reference. Protein bands were visualized with an ImageQuant LAS 4000 mini (General Electric Company; Boston, MA, USA).

### Chromatin immunoprecipitation quantitative PCR (ChIP-qPCR)

To do ChIP experiments, cells were plated into 15-cm dishes. After 42-h incubation, the drugs were added and incubated for additional 6 h. Then, the SimpleChIP® Enzymatic Chromatin IP Kit (#9003) (Cell Signaling Technology; Boston, MA, USA) was used to obtain DNA following the manufacturer’s procedure. Quantitative PCR was performed to measure the purified DNA with SYBR® Premix ExTaq™ (Tli RNaseH Plus). Primer sequences for amplifying the human *IDO1* gene promoter were 5′-ACGGGCAACTTGGTTTCTTC-3′ (forward) and 5′-CATGCAAGTCTGTGGTTCACT-3′ (reverse). The ChIP-qPCR primers were designed based on the H3K27 acetylation enrichment on the *IDO1* promoter region. The enrichment of H3K27 acetylation on the *IDO1* promoter in cells was obtained from the ENCODE website. Human α Satellite Repeat Primer (#4486) was purchased from Cell Signaling Technology (Boston, MA, USA).

### HPLC and cell number measurements

HPLC was used to determine the content of l-kynurenine following a modified method^35^. Cells were plated into 12-well plates and incubated for 24 h. The drugs were added and cells were incubated for additional 24 h. For the HPLC analysis, 500-μl supernatants were fully mixed with 200 μl of 30% (wt/vol) trichloroacetic acid to precipitate proteins. After centrifuging at 16,000 *g* for 5 min, the supernatants were filtered using a 0.22-μm filter membrane. Then, the resulting solution was injected into a column (LC1290, Agilent Technologies; CA, USA) to conduct HPLC. To identify and measure the content of l-kynurenine, the standard known content of l-kynurenine was used as an external reference to determine the retention time and the relationship between the content of l-kynurenine and the area under the curve of the HPLC spectrum. To exclude the influence of the reduction of cell number on the measurement of the l-kynurenine content, the cell number was measured by the sulforhodamine B^36^ assay after removing cell supernatants for HPLC analyses, and the data were expressed as the corresponding optical density values.

### Animal experiments

Female Balb/c nude mice (5–6-week-old) were obtained from the Shanghai Ling Chang Biological Technology Co., Ltd. (Shanghai, China). Human Ty-82 xenograft models were established following the previously reported method^37^. Mice with the established xenografts were given (*p.o*.) the BET inhibitor ABBV-075 daily at 0.5 mg/kg or vehicle. Tumor size was measured twice a week and body weight was measured daily. Tumor volume was calculated according to the previously described method^37^. After a 21-day continuous treatment cycle, mice were killed and the remaining xenografts were removed for western blotting and RT-qPCR. The animal experiments abided by the Guide for the Care and Use of Laboratory Animals (National Institutes of Health; Bethesda, MD, USA), and the care and use of the animals were approved by the Institutional Animal Care and Use Committee of the Shanghai Institute of Materia Medica.

### Statistical analyses

All data, if applicable, were presented as mean ± SD. Significant differences were determined by paired Student’s *t*-test (two tails) (Figs. 1–5) or group Student’s *t*-test (two tails) (Fig. 6). *p* *<* 0.05 was considered statistically significant.

## Supplementary information

Supplementary Figure S1

Supplementary figure legend

Supplementary Table S1

Supplementary Table S2

Supplementary Table S3
