# Supplementary figures and images for: Inhibition of the BET family reduces its new target gene IDO1 expression and the production of l-kynurenine
Source: Cell Death Dis. 2019 Jul 19;10(8):557. doi: 10.1038/s41419-019-1793-9 (PMC6642217; doi:10.1038/s41419-019-1793-9)

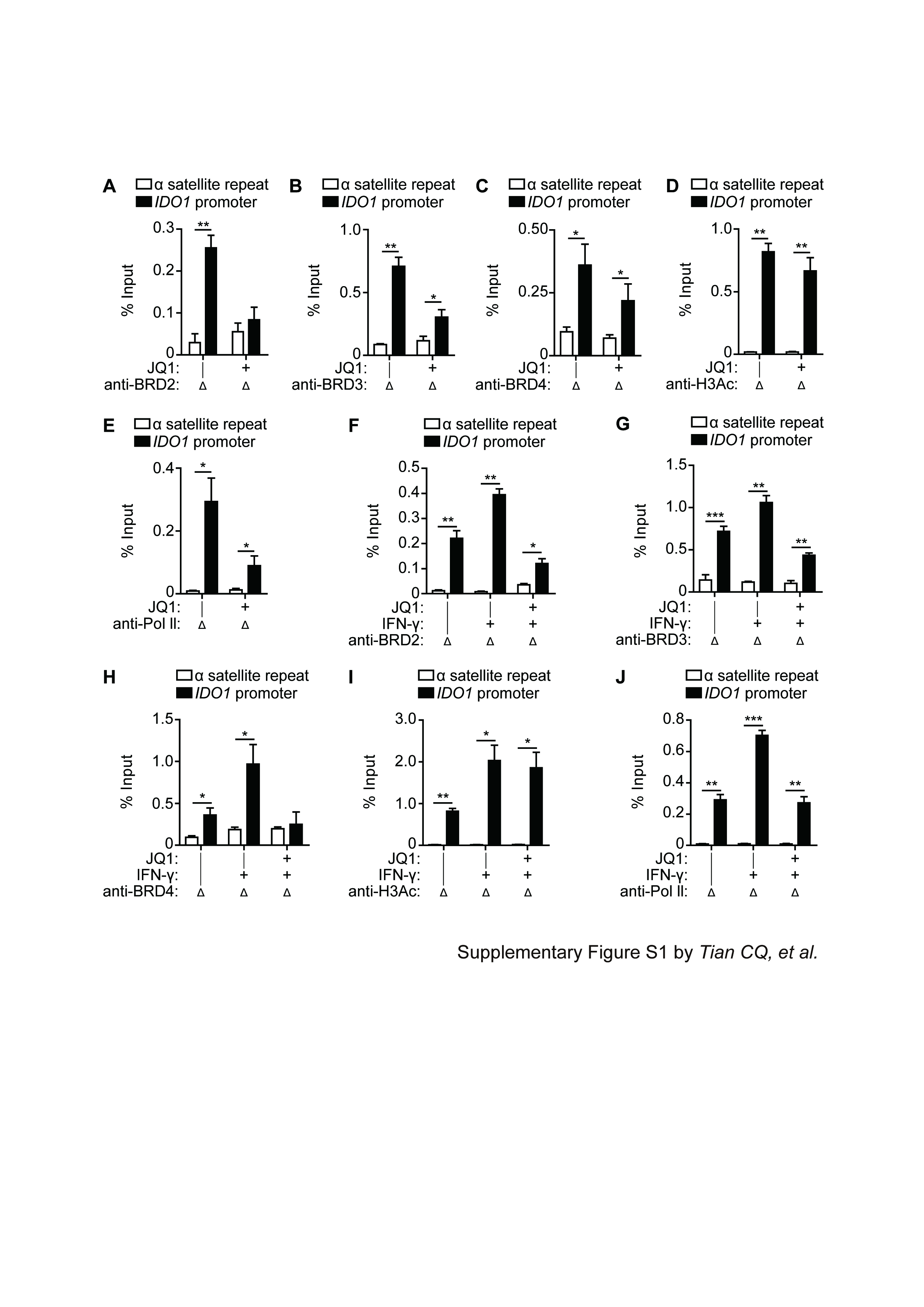

Supplement: Supplementary file 1 — Supplementary Figure S1 [file 41419_2019_1793_MOESM1_ESM.tif]
