# Supplementary material for: Inhibition of the BET family reduces its new target gene IDO1 expression and the production of l-kynurenine
Source: Cell Death Dis. 2019 Jul 19;10(8):557. doi: 10.1038/s41419-019-1793-9 (PMC6642217; doi:10.1038/s41419-019-1793-9)
Supplement: Supplementary file 2 — Supplementary figure legend [file 41419_2019_1793_MOESM2_ESM.docx]

**Supplementary figure legend**

**Supplementary Fig. S1 JQ1 selectively inhibits the binding of the BET family proteins to the promoter of *IDO1* and Pol II. A**-**E** SKOV3 cells were treated with or without 1 μM JQ1 for 6 h. The cells were subjected to ChIP analyses using antibodies against BRD2 (**A**), BRD3 (**B**), BRD4 (**C**), H3Ac (**D**) or Pol II (**E**). Their association with the *IDO1* promoter or the α satellite repeat was quantified by qPCR. The human α satellite repeat was used as a putative negative control. **F**-**J** SKOV3 cells were treated with IFN-γ (10 ng/ml), 1 μM JQ1 or both for 6 h. The association of BRD2 (**F**), BRD3 (**G**), BRD4 (**H**), H3Ac (**I**) or Pol II (**J**) with the *IDO1* promoter or the α satellite repeat was quantified by qPCR. The data were from 3 independent experiments and expressed as mean ± SD (Error bar); *p* < 0.05, *; 0.01, **; and 0.001, ***; +, treated with JQ1 or IFN-γ; Δ, detected with the indicated antibody.
